# Analysis of the association between the sex and disease courses of 132 consequent patients with HTLV-1-associated myelopathy/Tropic spastic paraparesis (HAM/TSP)

**DOI:** 10.1186/1742-4690-12-S1-P20

**Published:** 2015-08-28

**Authors:** Eiji Matsuura, Satoshi Nozuma, Ryuji Kubota, Shuji Izumo, Hiroshi Takashima

**Affiliations:** 1 Department of Neurology and Geriatrics, Kagoshima University Graduate School of Medical and Dental Sciences, Japan; 2 Department of Molecular Pathology, Center for Chronic Viral Diseases, Kagoshima University, Japan

## Objective

HTLV-1-associated myelopathy/tropical spastic paraparesis (HAM/TSP) is predominant in female. The course of disease differs by the individuals. The difference in the host's immune response between mother to child infection by breast feeding and an infection by a sexual intercourse may associate with the difference in prevalence between the sexes and in disease course of the patients. Hence, we analyzed the association among sex and clinical parameters of HAM/TSP to clarify the effect of an infection by a sexual intercourse on the pathogenesis of the disease.

## Methods

We reviewed the records of 132 patients with HAM/TSP who were admitted in series in the last 10 years in Kagoshima University Hospital. The cases were divided into the two subgroups, a group of the patients presenting rapid progression and a group of the patients presenting slow progression. Age, age of onset, duration of illness, an initial symptom, Osame's Motor Disability Score (OMDS), laboratory data and the type of disease course were evaluated. These data were tabulated according to sex and subgroup.

## Results

Of 132 consequent patients who had been diagnosed with HAM/TSP from 22 and 2012, 1 cases (75.8%) were female. There was no difference in age of onset, an initial symptom, the laboratory data and an incident rate of the patients with rapid progression, except but a level of protein in cerebrospinal fluid (CSF) between the sexes. Compared with the patients with slow progression, cell counts, level of protein and anti-HTLV-1 antibody titer were increased in the CSF of the patients with rapid progression, suggesting inflammatory process of the spinal cord in the patients with rapid progression. Interestingly, HTLV-1 PVL was significantly lower in patients with rapid progression than in those with slow progression (370 vs. 1,174 copies, p < 0.1) However, the association between the sex and rapid progression was not detected.

## Conclusions

We demonstrated no differences among the sex, onset age and the disease course of HAM/TSP. The reason why HAM/TSP is predominant in female still remains unknown. The results also suggest that factors other than the sex contribute to a disease course of HAM/TSP.

